# Efficacy of mesenchymal stromal cells and cellular products in improvement of symptoms for COVID‐19 and similar lung diseases

**DOI:** 10.1002/bit.27729

**Published:** 2021-03-27

**Authors:** Behnaz Banimohamad‐Shotorbani, Hekmat Farajpour, Farshid Sefat, Shiva Ahdi Khosroshahi, Hajar Shafaei, Saeed Heidari keshel

**Affiliations:** ^1^ Student Research Committee University of Medical Sciences Tabriz Iran; ^2^ Department of Tissue Engineering, Faculty of Advanced Medical Sciences Tabriz University of Medical Sciences Tabriz Iran; ^3^ Department of Tissue Engineering and Applied Cell Sciences, School of Advanced Technologies in Medicine Shahid Beheshti University of Medical Sciences Tehran Iran; ^4^ Department of Biomedical and Electronics Engineering, School of Engineering University of Bradford Bradford UK; ^5^ School of Engineering, Interdisciplinary Research Center in Polymer Science & Technology (Polymer IRC) University of Bradford Bradford UK; ^6^ Department of Medical Biotechnology, Faculty of Advanced Medical Sciences Tabriz University of Medical Sciences Tabriz Iran; ^7^ Department of Anatomical Sciences, Faculty of Medicine Tabriz University of Medical Sciences Tabriz Iran

**Keywords:** COVID‐19, extracellular vesicles, mesenchymal stromal cell, respiratory diseases, secretome

## Abstract

At the end of 2019, respiratory coronavirus diseases 2019 (COVID‐19) appeared and spread rapidly in the world. Besides several mutations, the outcome of this pandemic was the death up to 15% of hospitalized patients. Mesenchymal stromal cell therapy as a therapeutic strategy seemed successful in treatment of several diseases. Not only mesenchymal stromal cells of several tissues, but also their secreted extracellular vesicles and even secretome indicated beneficial therapeutic function. All of these three options were studied for treatment of COVID‐19 as well as those respiratory diseases that have similar symptom. Fortunately, most of the outcomes were promising and optimistic. In this paper, we review in‐vivo and clinical studies which have been used different sources of mesenchymal stromal cell, secreted extracellular vesicles, and secretome to improve and treat symptoms of COVID‐19 and similar lung diseases.

## Graphical abstract

The main event of COVID‐19 infection and MSC‐Based treatment, In step1, viruses are present in patient's lung and could infected others by dispersing of the virus particles (as an example by cough), then these particles enter into the body of healthy man (2), viruses bind to ACE2 receptor in cells of the respiratory system (3), symptoms could be different between patients from tolerable condition to intolerable states which the patient needs to be hospitalizing. These patients can make other people sick (4). MSC‐Based therapies include MSCs, MSC‐EVs, and MSC‐secretome (5) that are from four main sources including, adipose, bone marrow, menstrual blood, and umbilical cord (6). Results show the potential of these treatments in COVID‐19 recovery (7). The recovered body could be infected and receive viruses again (8).

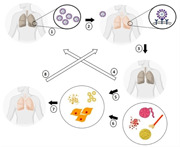

## 1 INTRODUCTION

At the end of 2019, several cases of acute pneumonia‐like respiratory diseases of unknown origin with some symptoms including fever, cough, and shortness of breath were reported in Wuhan, Hubie province of China. The etiological agent was characterized as a novel coronavirus due to the whole‐genome analysis results which shown to be transmitted from human‐to‐human by either droplets or direct contact. International Committee of Taxonomy of Viruses officially named the virus severe acute respiratory syndrome coronavirus 2 (SARS‐CoV‐2). This respiratory disease which meanwhile was termed coronavirus diseases 2019 (COVID‐19) were spread rapidly by the human to human transmission, cause an outbreak worldwide with considerable morbidity rate and mortality, which forced WHO to officially classified it as a pandemic on March 11th, 2020 (Pourjabbar et al., 2020).

Structural analysis suggested that mutations in the spike glycoprotein (S protein) and also nucleocapsid N protein of the bat SARS‐like coronavirus were responsible source of COVID‐19 (Dawood, 2020). It seems that the virus has some several new mutations during replication because of RNA‐dependent RNA polymerases errors (Lau et al., 2020). Lately, Koyama et al. reported 5775 different variants of the SARS‐CoV‐2 genome including synonymous, missense, deletion, and in‐frame insertion mutations (Koyama et al., 2020). The effect of these mutation may be deferent. For example, previously it was not proved that dominant variant of S protein D614G lead to more severe disease (until March 2020) (Grubaugh et al., 2020) but at July 2020, it was reported that this mutation could increases viral infectivity and transduction. Proteolytic cleavage resistance of the G614 variant was proposed as a probable mechanism. D614G mutation refers to aspartate to glycine mutation (Daniloski et al., 2020; Hu et al., 2020). As a second example, the binding affinity of protein S for ACE2 could increase remarkably by the single N501T mutation (Shereen et al., 2020; Wan et al., 2020). Also, it was suggested that the accumulation of mutations in two genes (the S gene and the accessory gene 5a) could lessen pathogenicity and mortality of infectious bronchitis coronavirus (Zhao et al., 2019). Beside several mutations, it is suggested that mortality is mainly related to the age of patients (Qu et al., 2020) and some diseases including serious heart conditions, chronic lung disease, obesity, diabetes, and high blood pressure (Koyama et al., 2020). Mortality of hospitalized COVID‐19 patients was about 5%–15% (Qu et al., 2020).

Mesenchymal stromal cells (MSCs) not only showed regenerative potential but also become a therapeutic option for several diseases (Uccelli & de Rosbo, 2015), such as orthopedic, cardiac, neurological and graft versus host disease (Lukomska et al., 2019). MSCs as multipotent cells are known for self‐renewing and differentiating into adipogenic, osteogenic, and chondrogenic lineages (Taghavi‐Farahabadi et al., 2020). MSCs can be found in connective tissue and organ stroma, including adipose tissue, bone marrow, umbilical cord blood, Wharton's jelly placenta, dental pulp, menstrual blood, periodontal ligament, amniotic and other tissues (Atluri et al., 2020; Gentile & Sterodimas, 2020; Wang, 2020). In 1995, MSCs were used as a cellular pharmaceutical agent in human cases (Lazarus et al., 1995) and up to now, they do not indicated adverse events in systemic administration (Poulos, 2018; Rajarshi et al., 2020). It has reported that the most prevailing source in recent clinical trials were adult bone marrow mesenchymal stromal cells (BM‐MSC), adipose tissue derived mesenchymal stromal cells (AT‐MSC), umbilical cord tissue mesenchymal stromal cells (UC‐MSC) and placental cells respectively (Galipeau & Sensébé, 2018). MSCs could secrete some antimicrobial molecules which could reduce feeling pain (Rogers et al., 2020). In‐vitro studies showed low expression of major histocompatibility complex‐I (MHC‐I) and no expression of MHC II or costimulatory molecules B7‐1, CD40, or B7‐2 in MSCs which leads to the immune‐evasively (Rogers et al., 2020).

It is proved that human MSCs shows heterogeneity in the quality, so their products depend on the donor, isolation procedure, and culture methods (Lukomska et al., 2019). The Food and Drug Administration (FDA) standards should be considered in labs that are the source of stromal cells (Atluri et al., 2020). The isolation source of MSC affects the gene expression like genes that are related to cell adhesion molecules (Shotorbani et al., 2017), HLA molecules (Rogers et al., 2020)), transcription factors, differentiation potential (Gebler et al., 2012), as well as functional differences. Therefore, Yen et al. (2020) suggested that “not all MSCs are equal.”

In this paper, we aim to review and compare different sources of MSC and MSC secreted extracellular vesicles (MSC‐EVs) in clinical and preclinical treatment of Coronavirus disease and its related lung diseases with similar symptoms.

### 1.1 COVID‐19 pathology, and immune response in a glance

Since December 2019, novel COVID‐19 initiated from China and after a few months, many people all over the world were contaminated (Öztürk et al., 2020). SARS‐CoV‐2 as one of the coronaviridae could induce respiratory diseases. Following infection, to kill the virus, immune system become over‐activated and caused cytokine storm (secretion of large volumes of inflammatory cytokines) that could lead to organ damage, air exchange dysfunction, edema, acute respiratory distress syndrome (ARDS), acute cardiac injury, pulmonary damage induced by ARDS, secondary infection and even death (Atluri et al., 2020; Taghavi‐Farahabadi et al., 2020).

SARS‐CoV‐2 is able to encode several structural proteins due to several open reading frames including E (envelope), M (matrix), S (spike), and N (nucleocapsid). The S protein could bind to the surface of human cells which express angiotensin‐converting enzymes 2 (ACE2) in the human respiratory tract epithelium (alveolar cell type II), cardiopulmonary tissues, and some hematopoietic cells (monocytes and macrophages) (Moore & June, 2020; Taghavi‐Farahabadi et al., 2020). In facing viral infections, efficient immune responses caused by interferons I (IFN‐I) of innate immune cells and consequently by T helper 1 (Th1) and cytotoxic T (CT). Virus genome can be identified by several pattern recognition receptors (PRRs) once it enters the lung cell. Retinoic acid‐inducible gene I, toll‐like receptor‐3 (TLR‐3), TLR7‐9, cyclic GMP‐AMP synthase (cGAS), and melanoma‐differentiation‐associated gene 5 are some examples of PRRs and then secretion of IFN‐I and inflammatory cytokines initiated. In patients with COVID‐19, the proliferation of the virus causes cytokine storm which is the crucial reason of ARDS in COVID‐19. Cytokine storm includes interleukin‐2 (IL‐2), IL‐7, IL‐10, interferon‐γ‐inducible protein 10, granulocyte‐colony stimulating factor, tumor necrosis factor‐alpha (TNF‐α), macrophage inflammatory protein‐1 alpha, and monocyte chemo‐attractant protein‐1. Inflammatory cytokines were made by pathogenic T cells after activation. Afterward, migration of inflammatory monocytes and other leukocytes, such as neutrophil to the lung were completed. This immune cell activation could lead inflammation that may induce pulmonary damage and some complications like pneumonia, ARDS, loss of lung function, and even death. It seems that suppression of cytokine storm as a reason of ARDS that leads to death, may reduce the inflammation and lung damage (Taghavi‐Farahabadi et al., 2020). Immune responses to the body once COVID‐19 virus contagiously affected the new patient to kill the virus and inhibit the advancement to difficult stages, demands the primary start of a specific adaptive immune response (Rao et al., 2020). There are diverse complicated mechanisms are involved in ARDS, targeting a single pathway or mediator as a therapeutic approach which cannot be beneficial enough. Consequently, targeting different aspects of immune‐pathogenesis and related damages seems to be essential (Taghavi‐Farahabadi et al., 2020). In the following parts, MSC administration in COVID‐19 and its related lung diseases will discuss to compare outcomes of different MSC sources to realize whether there is any relation between cell source and successful ideal strategy or not.

### 1.2 MSCs and secreted extracellular vesicles

Comparing BM‐MSCs to AT‐MSCs and AT‐MSCs, not only indicates lower expression of HLA‐I (Rogers et al., 2020) but also, shows higher quantity of cells (Gentile & Sterodimas, 2020; Kawecki et al., 2018) with better in vitro proliferation (Kawecki et al., 2018). Furthermore, AT‐MSC potency could be preserved with the age of the donor, unlike BM‐MSCs (Rogers et al., 2020). Additionally, UC‐MSC population especially Wharton jelly (as one of the wealthy origins) is higher than BM‐MSCs (Atluri et al., 2020). Other advantageous of UC‐MSCs are higher plasticity, probably more potency, faster doubling times, and being scalable (that will be important in the great population of COVID‐19 cases) with the noninvasive obtaining process (Atluri et al., 2020). Menstrual blood derived MSCs (men‐MSCs) of young healthy women proliferated twice faster than BM‐MSCs. Men‐MSCs express markers of MSCs and some embryonic markers like Nanog and SSEA‐4. Different studies showed the differentiation potential of Men‐MSCs into bone, adipose, cartilage, neural, cardiac, and hepatic cells (Khoury et al., 2014). Widespread advancement were applied to explain the immune characteristics of MSCs and immune effects of Men‐MSCs which required further investigations (Khoury et al., 2014). AT‐, BM‐ and UC‐MSCs could inhibit B cell, T cell, and natural killer (NK) cell‐mediated immune response through inhibiting acquisition of lymphoblast features, triggering, and altering the protein expression with an critical role in immune response, but AT‐MSCs do it in earlier phase and higher than UC‐ or BM‐MSCs (Ribeiro et al., 2013). Notwithstanding the similarities some differences between MSCs were found, therefore, selecting the suitable MSC source for therapeutic or experimental plans became more important (Ribeiro et al., 2013).

For instance, the mortality in preclinical studies of acute lung injury models, UC‐ and BM‐MSCs seemed more effective than AT‐MSCs (Yen et al., 2020).

Micro‐vesicles (MVs) and exosomes are extracellular vesicles (EVs) with 0.03‐1 μm size that secreted from all cells (Muraca et al., 2020). Published evidence demonstrated the interesting events of the paracrine release of MSC‐EVs that contain various regulatory microRNAs (miRNAs), messenger RNAs (mRNAs), bioactive proteins, organelles, such as mitochondria, and some other compounds with the regulatory role. Interestingly, these MVs and exosomes could be used rather than direct cell substitution (Abraham & Krasnodembskaya, 2020; Lukomska et al., 2019). MSC‐EVs are similar to MSCs not only in round shape, but also in mesenchymal marker expression and lacked the expression of swine leukocyte antigens I and II (Khatri et al., 2018). Secreted MSC exosomes showed both typical markers of the exosome surface (including CD81 and CD9) and some adhesion molecules of MSC membrane (like CD73, CD44, and CD29) (Yu et al., 2014). It was shown that secretion of microparticles (that are enriched for pre‐miRNA by MSCs) could facilitate miRNA‐mediated intercellular communication (Chen et al., 2010).

The most general way for exosome isolation is ultracentrifugation that gives highly enriched exosomes. This method usually used in combination with sucrose cushions or sucrose density gradients. The centrifugal forces remove larger particles and cells and finally exosomes would precipitated (Yu et al., 2014). It was reported that this method could cause the isolation of heterogeneous MVs, such as smaller exosomes (Monsel et al., 2015). High‐performance liquid chromatography is another method that was used rarely due to the complexity. It includes two filtration steps, low‐gravity centrifugation, purification (by exclusion chromatography), and final centrifugation step. Also, exosome could be isolated using ultrafiltration based on the size. Comparing to ultracentrifugation, this method requires less time and does not demand specific devices. Furthermore, nowadays several kits for exosome isolation are produced by various companies. It was reported that storing exosomes at 37°C or 4°C may led to size reduction after some days, while at −20°C they can be stored for a long time without any change in the size and therefore, storage situation seems to be an important issue (Yu et al., 2014).

### 1.3 MSC and MSC‐EVs in lung diseases especially in COVID‐19

Orleans et al. thought that although BM‐MSC injection is useful in some diseases, such as spine and joint, but they would not likely be ideal for coronavirus treatment which is a serious systemic illness (Atluri et al., 2020). On the other hand, in a retrospective study was reported that cautious MSCs therapy could be a hopeful treatment of severe COVID‐19 (particularly in patients with coronary heart disease or metabolic acidosis it should be done cautiously) (Chen, Shan, et al., 2020). Several cell‐based therapies have been done for issues such as pulmonary diseases (Atluri et al., 2020; Behnke et al., 2020; Iyer et al., 2009). It was reported that the intravenous (IV), the intra‐alveolar, and inhalation route are efficient for cell delivery. Homing of MSCs happens in damaged organs. Survival can be reduced due to some other injuries in patients with acute lung injury, IV delivered MSCs which could cause better systemic treatment in the lung and other organs (Chrzanowski et al., 2020; Matthay et al., 2010). Also, after IV administration of MSCs, early observation indicated that highest amount of cell were present in the lungs, liver and spleen (Leibacher & Henschler, 2016). Also, improvement of myocardial infarction by IV administration in mice was reported by Lee et al. They believed that it can be due to embolized lung cell activation that leads to the secretion of Anti‐inflammatory Protein TSG‐6 (Lee et al., 2009). MSC accumulation in the lung is the result of IV administration and was followed by secretion of several paracrine factors (Lee et al., 2009; Shetty, 2020). Improvement of lung function, counteracting of fibrosis, and protecting epithelial cells of alveolar are notable effects of these factors (Shetty, 2020). Also pulmonary settlement of MSCs by IV administration could lessen over activation of the immune system and support tissue regeneration by improving microenvironment of the lung. IV administration of MSCs in COVID‐19 patients especially in aged people with severe pneumonia considers as an effective and safe treatment (Esquivel et al., 2020; Leng et al., 2020; Shetty, 2020). In a pilot, clinical study MSC‐exosomes were administered via the inhalation route. Comparing IV injection, inhalation administration prevents exosome aggregation in the injured microcirculation (Chrzanowski et al., 2020). Although, IV route is the most common way but it was suggested that choosing the route of administration should consider the patient's circumstances. On the other hand, it was proposed that the inhalation route for chronic lung disease is a more direct method with a lower incidence of unfavorable results. Beside this for using the inhaled route for treatment of COVID‐19 patient's hospital environment must be properly managed (Chrzanowski et al., 2020).

Signaling molecules of damaged tissue and their receptors on the MSCs helps the MSC homing process (Squillaro et al., 2016). Although, MSC become disappear in 24–48 h, they can persist for a longer time in inflamed or damaged lungs (Khoury et al., 2020). Additionally, they could preserve the vascular endothelial and alveolar epithelial barrier function in ARDS animal models (Zhao & Zhang, 2020). It was shown that using MSC cause promising outcomes in ARDS treatment (Taghavi‐Farahabadi et al., 2020). The ability of MSCs to suppressing those immune responses that are exaggerated in tissue repair or regeneration, called immune‐modulatory. Therefore, it was suggested that in the absence of inflammatory responses, MSCs have the potential to manage severe symptoms of COVID‐19 infection in patients and reduce lung injury (Rao et al., 2020; Taghavi‐Farahabadi et al., 2020). Latterly Wilson et al. showed that treatment of (nine) patients with ARDS by allogeneic MSC does not cause pre‐specified adverse effects, such as cardiac arrhythmia, hypoxemia, and ventricular tachycardia (Chen, Yu, et al., 2020; Wilson et al., 2015).

In an excessive immune response, MSCs secrete various soluble agents including angiogenic growth factors (Khoury et al., 2020), antimicrobial peptides (Khoury et al., 2020; Krasnodembskaya et al., 2010), EVs (Khoury et al., 2020), transforming growth factor (TGF), Nitric oxide (NO), soluble IL‐6, indoleamine 2 3‐dioxygenase (IDO) HLA‐G5, and beta Prostaglandin E2 (PGE2) (Rao et al., 2020). These agents reduce genesis of interleukin 17 and interferon‐γ. Basically, these could prevent the cytotoxic CD8+ cell activation through diminishing direct damage to the lung parenchyma (Rao et al., 2020). Also, PGE2 pathways decreased maturity and activity of dendritic cells (DCs) therefore, IL‐10 and TNF‐α reduced (anti‐inflammatory effect). This could activate Treg cells and additional enhancement in IL‐10 production. NK cells could be suppressed via the excretion of these soluble agents as well as contact‐mediated communication with MSCs. On the other hand, MSCs could enhance mobilization and reduce the chemotaxis of neutrophils (Rao et al., 2020). IV transplanted MSCs could reach the lungs speedily (Wang, 2020). Unfortunately, in a recent report, it has claimed that the outcome of IV injection of MSCs is nonuniform differentiation and embolise in the lungs that may lead to epithelial damage (Poulos, 2018). In other study, IV infusion of MSC therapy introduced safe and efficient for COVID‐19 pneumonia, even in aged patients with severe pneumonia (Shetty, 2020). Rajarshi et al. reported anti‐inflammatory and immune‐modulatory of MSCs in clinical studies related to respiratory diseases (Rajarshi et al., 2020).

Lately, not only other cell types, but also MSC‐EVs or conditioned media (CM) have been investigating largely for COVID‐19 treatment in China (Khoury et al., 2020). Therapeutic effects of MSC‐EV was indicated in animal models of lung injury, such as viral pneumonia (Khatri et al., 2018), severe bacterial pneumonia (Monsel et al., 2015), and hyperoxia (Braun et al., 2018; Porzionato et al., 2019; Willis et al., 2018). Also, in the murine model of lung disease, reduced lung weight gain following perfusion and ventilation, enhanced clearance of alveolar fluid, and developed hemodynamic and airway parameters were observed after MSC‐EVs administrating that leads to the rehabilitation of marginal donor human lungs. The prevention effect of MSC‐EVs on fibrosis improvement was similar to their cell source. It has believed that there are important limitations to use MSC‐EVs as therapeutic tools that some are shared with their cell sources such as culture situations or variability of tissue origin. MSCs of different sources may behave differently in differentiation or immune‐suppressive, and still there are no comparative studies on different sources of MSC‐EVs (Muraca et al., 2020).

### 1.4 Immune‐modulatory of MSCs

MSCs could be used for treatment of different disease due to their properties (Taghavi‐Farahabadi et al., 2020). First in 2000, immune modulatory of MSCs was demonstrated (Squillaro et al., 2016). Altering the immune cell function and modulating the immune response can be done by MSCs. In the animal model of LPS‐induced ARDS, inflammation suppressing, and decreasing inflammation‐induced lung damage caused by MSCs. Macrophages are affected by PGE2 secreted by MSCs. MSCs also decrease inflammatory cytokine generation and enhance IL‐10 production. Reduction of neutrophil recruitment to the lung and inflammatory cytokine generation occurs by IL‐10. Also, enhanced Treg cells and altered macrophage phenotype (from M1 to M2) carried out by MSCs too (Taghavi‐Farahabadi et al., 2020). Low expression of MHC makes MSCS nonimmunogenic and satisfied for allogenic therapeutic interventions without HLA matching. In several autoimmune diseases, different sources of MSCS have been employed for immune modulation (Le Blanc & Mougiakakos, 2012; Rao et al., 2020). Activated NK cells could inactivate or lyse MSCs. This event could be controlled by priming the MSCs with interferon‐γ before transfusion (Rao et al., 2020). Long‐term immune‐modulatory of the MSC sustained by continuous cytokine generation (Metcalfe, 2020). Inflammation regulatory mechanisms of MSCs include macrophage encouragement to anti‐inflammatory phenotypic polarization, DCs maturation, proliferation, and differentiation inhibition in B lymphocytes, and improving the recruitment of Treg cells, such as CD4+CD25+FoxP3+ T lymphocytes and CD8+CD28− T lymphocytes (Wang, 2020).

Additioanlly, MSCs not only arrest cell division of NK cells, B cells, and DCs but also affect some other functions of immune cells. These effects include maturation and antibody secretion of B cells, cytotoxicity, and cytokine secretion of T and NK cells, maturation, activation, and antigen presentation of DCs (Uccelli et al., 2007).

### 1.5 Lung regeneration

MSCs could inhibit lung cell apoptosis and encourage them to regenerate especially alveolar cell II. Providing some growth factors such as vascular endothelial growth factor (VEGF), hepatocyte growth factor (HGF), and keratinocyte growth factor (KGF). Angiopoietin‐1 secretion by MSCs could restore the permeability of epithelial protein (Matthay et al., 2010; Taghavi‐Farahabadi et al., 2020). In a model of lung fibrosis, it was shown that MSC administration could decrease both collagen deposition and inflammation (Uccelli et al., 2007). Micro‐vesicle transferring of MSCs improve macrophage phagocytosis capacity (Taghavi‐Farahabadi et al., 2020). Additionally, evidence showed that MSC‐based treatments could inhibit collagen accumulation and alveolar collapse (Chen, Yu, et al., 2020).

### 1.6 Antiviral activity

Some studies reported about antiviral activity of MSCs (Yang et al., 2015). Yang et al. infected MSCs by gamma herpes virus. MSCs sense virus DNA through cGAS and then initiate the STING‐TBK1 signaling pathway to restricting replication. This pathway generates IFN‐γ and also is responsible for the antiviral function of MSCs in the IFN‐γ independent manner (Taghavi‐Farahabadi et al., 2020; Yang et al., 2015). Furthermore, some other investigation demonstrated that IDO expression of MSCs may be responsible for the antiviral behavior too (Taghavi‐Farahabadi et al., 2020). Meisel et al. showed that IDO‐positive MSCs triggered by inflammatory cytokines act as antimicrobial effectors against pathogens like viruses (Meisel et al., 2004; Taghavi‐Farahabadi et al., 2020). On the other hand, Meisel et al. reported that IDO expression of MSCs could be stimulate by FN‐γ and also could reduce the HSV‐1 and CMV replication (Meisel et al., 2011; Taghavi‐Farahabadi et al., 2020).

**Figure 1:**
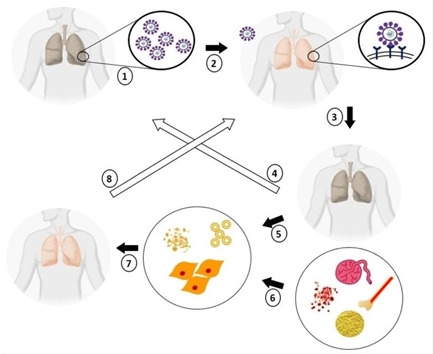
The possible inhibitory role of exosome on cytokine storm (Kadriyan et al., 2020,) [Color figure can be viewed at wileyonlinelibrary.com]

Also, viral resistance of MSCs was reported previously (Khoury et al., 2020) and another study demonstrated that MSC won't be infected by Covid‐19. As MSCs are ACE2 negative initially and therefore, during the follow‐up they did not differentiate or shift to ACE2 positive (Metcalfe, 2020). Beside antiviral activity, in‐vitro and in‐vivo investigations showed MSC innate antimicrobial features that could increase macrophage removal of bacteria that confirm innate antimicrobial attributes (Rogers et al., 2020) (Figure 1).

### 1.7 Potential of different sources of MSC, MSC‐EVs and secretome in lung disease especially COVID‐19

MSC therapy of very sickest COVID‐19 patients was allowed by the US FDA under the clause for expanded access compassionate (Soni & Srivastava, 2020). In a pilot clinical trial, the effect of MSC transplantation in COVID‐19 was evaluating. All cases had a high fever, low oxygen saturation, and shortness of breath. Results represent no adverse events, developed pulmonary function, lowered inflammatory cytokines, enhanced peripheral lymphocytes, shifting to DCs and CD4+ T cells, and increasing IL‐10 (Leng et al., 2020; Metcalfe, 2020; Qu et al., 2020). The source of the cells was not reported in this study. According to a systematic review, UC‐MSCs and BM‐MSCs showed further decreasing in mortality than AD‐MSCs in in‐vivo acute lung injury models (Leng et al., 2020). Also, ventilation‐induced lung injury could be recovered by paracrine mechanism of intra‐tracheal (IT) and IV MSC administration too (Curley et al., 2013).

Effect of MSC administration on mice shows salubrious effect on reduction of sepsis‐related morbidity and mortality. They assumed that immune‐modulatory effect may be carried out by paracrine mechanisms. Unfortunately, tissue source of MSCs was not mentioned in this study (Mei et al., 2010). In following parts, MSCs administering for lung diseases with similar symptoms to COVID‐19 are classified and discussed by origin.

December 27, 2020, result of searching on https://clinicaltrials.gov/ via key words, MSC and COVID‐19 led to 276 studies. The most numbers of trials are related to North America. All related results were gathered in Table 1.

**Table 1:** Distribution of mesenchymal stem cell (MSC) and COVID‐19 for up to December 27th, 2020

**Countries**	**Mesenchymal stem cell | COVID‐19**	**COVID19 | MSC**	**Mesenchymal stem cell | Coronavirus infection**	**MSC | Coronavirus infection**
Africa	‐	13	‐	8
Central America	‐	1	‐	‐
East Asia	11	16	8	12
Europe	10	81	2	27
Middle East	3	31	2	10
North America	20	83	8	32
North Asia	4	6	2	2
Pacifica	1	7	‐	4
South America	2	26	1	8
South Asia	3	6	2	3
Southeast Asia	2	8	1	5
Total	68	276	31	102

Abbreviation: COVID‐19, coronavirus disease 2019.

### 1.8 AT‐MSC

Gentile et al. thought that AT‐MSCs may be the most important representative of MSCs (Gentile & Sterodimas, 2020). In 2014, allogeneic AT‐MSCs therapies reported as a feasible and safe approach for ARDS treatment (Zheng et al., 2014). In an in‐vivo study, it was shown that AT‐MSCs can inhibit the nuclear factor‐κB signaling pathway, reduce pulmonary inflammation, decreasing pulmonary proinflammatory factor expression and also, reverse the pulmonary fibrosis process of induced by amiodarone (Wang, 2020). Also, they decrease ventricular systolic pressure, smooth muscle cell proliferation, lung tissue collagen fiber content, CD68+ macrophages, Bcl‐2, and interleukin‐6 in pulmonary arterial hypertension. However, plasma VEGF and procaspase‐3 enhanced. Furthermore, the pulmonary artery may cause dampening of the endothelial–mesenchymal transition (de Mendonça et al., 2017). Another investigation showed that immune‐modulatory effect of AT‐MSC on DC differentiation is more than BM‐MSCs (Rogers et al., 2020).

Another research group reported the efficiency and safety of human AT‐MSCs with related stromal vascular fraction (SVF) portion which were used in the immune‐mediated inflammatory diseases and autologous regenerative therapies. SVFs and AT‐MSCs causing secretion of proangiogenic factors (VEGF, platelet‐derived growth factors [PDGF]), immune‐modulating proprietors (TGF‐β1, HGF, interferon‐γ [INF‐γ]), promotion of the vascularization, and preparing physical extracellular matrix conductance to promote sprouting of endothelial cells (Gentile & Sterodimas, 2020). Result of clinical and preclinical studies of each type of MSCs are summarized in Table 2.

**Table 2:** Summarized main affects of administration of several sources of MSCs in COVID‐19 associated diseases in clinical and preclinical studies

**In vivo studies using MSCs in lung diseases**			
**Disease/(animal)**	**Type and source of MSC/quantity/administration method/period of fallow up (for clinical studies)**	**Results/suggestion**	**References**
Monocrotaline‐induced pulmonary arterial hypertension/(male Wistar) rat	AT‐MSC/10^5^ cell/ntravenous	‐ Collagen fiber content of lung tissue^b^ ‐ Smooth muscle cell proliferation^b^ ‐ CD68+ macrophages, interleukin‐6 expression^b^ ‐ Expression of procaspase‐3 and plasma VEGF^d^ ‐ PDGF expression: No changes‐ Hemodynamics^d^ (by mitigating lung vascular remodeling)‐ Endothelial–mesenchymal transition of MSCs in the pulmonary artery	de Mendonça et al. (2017)
Intraalveolarendotoxin‐induced ALI/(nonimmuno‐suppressed C57BL6) mice	BM‐MSC/not given/intratracheal route	‐ Pulmonary edema^b^ ‐ Histologic lung injury^b^ ‐ Proinflammatory cytokines^c^ ‐ Anti‐inflammatory cytokines (IL‐10 and IL‐13)^d^ ‐ Survival^e^	Gupta et al. (2007)
Endotoxin‐induced lungedema and inflammation/mice	BM‐MSC/$5\times 10^{5}$ Cells/Intravenous	‐ Lung injury^a^ ‐ Edema^a^‐ Inflammatory responses (systemic and local)^b^ ‐ MIP‐1α, IL‐1β, IL‐12, RANTES, and IL‐6^b^ ‐ IL‐10 concentrations: unaffected‐ If BM‐MSC contact lung cells could effect on MIP‐1 α and RANTES stronger than the condition in which contact was not feasible.	Xu et al. (2007)
E. coli endotoxin‐induced acute lung injury in the ex vivo perfused human lung	hBM‐MSC/$5\times 10^{6}$ orMSC‐CM	‐ Clearance rate of alveolar fluid^d^ (to a normal level)‐ Lung vascular permeability and extravascular lung water returned to normal levels.	Lee et al. (2009)
Myocardial infarction/mice	hBM‐MSC/$2\times 10^{6}$ hMSCs/Intravenously	‐ Anti‐inflammatory protein TSG‐6^d^ ‐ Expression of the anti‐inflammatory factor TNF‐α‐induced protein 6 (TNAIP6 or TSG‐6) by cells that trapped as emboli in lungAfter myocardial infarction, Intravenous hMSCs, but not hMSCs transduced with TSG‐6 siRNA:‐ Inflammatory responses and infarct size^b^ ‐ Cardiac function^d^. Intravenous administration of recombinant TSG‐6:‐ Inflammatory responses and infarct size^b^	Lee et al. (2009)
Bleomycin (BLM)‐induced inflammation/mice	BM‐MSC/$5\times 10^{5}$/injection into the jugular vein	‐ Inflammatory^b^ ‐ Collagen deposition within lung tissue^b^ ‐ Homing to lung in response to injury‐ Adopt an epithelium‐like phenotype	Ortiz et al. (2003)
Ventilator‐induced Lung Injury/rat	BM‐MSC/$4\times 10^{6}$ intratracheal MSCs; 300 µl intratracheal conditioned medium; $4\times 10^{6}$ intravenous MSCs	Intratracheal MSC therapy:‐ Repair after ventilation‐induced lung injury^d^ ‐ Arterial oxygenation^d^ ‐ Total lung water^b^ ‐ Lung inflammation^b^ ‐ Histologic injury^b^ ‐ Restoring lung compliance‐ Quantity of alveolar tumor necrosis factor‐α and interleukin‐6^b^ ‐ Bronchoalveolar lavage KGF^d^ ‐ Lymphocytes in the alveolar fluid^d^. Intratracheal MSCs with conditioned MSC medium:‐ Lung repair after injury^d^ ‐ Alveolar inflammatory cell infiltration^b^ intravenous MSC administration:‐ Amount of bronchoalveolar lavage IL‐10^d^ ‐ Epithelial and endothelial repair^d^ ‐ Lymphocytes in the alveolar fluid^d^. The efficiency of intravenous and intratracheal MSC administration was similar	Curley et al. (2013)
Bronchopulmonary dysplasia (BPD) and emphysema/rat	BM‐MSCs/$1\times 10^{5}$ cells per animal/intratracheal delivery	‐ Survival^d^ ‐ Exercise tolerance^d^ ‐ Alveolar and lung vascular injury^b^ ‐ Pulmonary hypertension^b^ ‐ Engrafted BM‐MSCs coexpressed the AEC2‐specific marker surfactant protein C. ‐ BM‐MSCs prevent arrested alveolar and vascular growth in part through paracrine activity.In vitro, BMSC‐derived CM:‐ O_2_‐induced AEC2 apoptosis^a^‐ Accelerated AEC2 wound healing‐ Endothelial cord formation^d^	van Haaften et al. (2009)
BPD/murine	BM‐MSC/$5\times 10^{4}$/intravenous	‐ Alveolar loss^b^ ‐ Lung inflammation^b^ ‐ Pulmonary hypertension^a^. Injection of bmsc‐cm:‐ Vessel remodeling^a^‐ Alveolar injury^a^‐ Normal alveolar numbers at Day 14 of hyperoxia‐ Lung neutrophil and macrophage accumulation^c^ ‐ Macrophage stimulating factor 1 and osteopontin^d^	Aslam et al. (2009)
Sepsis (clinical syndrome of severe systemic inflammation precipitated by infection)/(C57Bl/6 J) mice	BM‐MSC/$2.5\times 10^{5}$/intravenous injection	‐ Mortality^c^ ‐ Systemic and pulmonary cytokine^b^ ‐ Acute lung injury^a^‐ Organ dysfunction^a^‐ Inflammation and inflammation‐related genes expression (such as IL‐10, IL‐6)^b^ ‐ Expression of genes involved in promoting phagocytosis and bacterial killing^d^ ‐ Phagocytotic activity of the host immune cells^d^ ‐ Bacterial clearance^e^	Mei et al. (2010)
ALI induced bylipopolysaccharide/mice	BM‐MSC transduced with the *Ang1* gene/$1\times 10^{5}$/intravenously (jugular vein)	‐ The expression of Ang1 protein in the recipient lungs^d^ ‐ Lung histopathology^d^ ‐ The histopathological and biochemical indices^d^ ‐ Pulmonary vascular endothelial permeability^b^ ‐ Recruitment of inflammatory cells into the lung‐ MSCs and Ang1 have a synergistic role in the treatment of LPS‐induced lung injury	Xu et al. (2008)
Clinical studies using MSCs in COVID‐19 associated lung diseases			
Acute respiratory distress syndrome (ARDS) induced by epidemic influenza A (H7N9) Infection	allogeneic Men‐MSCs/1 million per kilogram of body weight/intravenousinfusion/Up to 5 years	‐ The mortality^b^ ‐ No harmful effects after 5 years‐ The procalcitonin level^d^ ‐ The serum creatinine level^b^ ‐ Level of prothrombin time (PT)^e^ ‐ Creatine kinase^e^ ‐ Upregulation of hemoglobin‐ Downregulation of PTR adiologic changes: ‐ Linear fibrosis, air bronchogram, bronchiectasia, isolated areas of pleural thickening, ground ‐glass opacities, and hydrothorax. MSC‐based therapy suggested as alternative for COVID‐19 treatment	Chen, Hu et al. (2020)
SARS‐CoV‐2 infection	Men‐MSCs/1 million per kg body weight/intravenous infusion/follow up 1 week after discharged from hospital	‐ The immune indicators (lymphocytes)^d^ ‐ Inflammatory indicators (such as IL‐6, IL‐10, TNF, and IFN)^b^ ‐ Mitigated symptom and being discharged (of two patients after 3 weeks MSC therapies)‐ Presenting anti‐inflammatory function by MSCs (suppressing, RANTES, GM‐CSF, MIG‐1g, MCP‐5, Eotaxin)	Chen, Yu, et al. (2020)
COVID‐19/case report	hUC‐MSC/(three) intravenous infusions of $5\times 10^{7}$ hUC‐MSC	‐ Circulating T cell counts^d^ (returned towards normal levels)‐ No obvious side effects‐ Pneumonia was greatly relieved	Liang et al. (2020)
severe COVID‐19	hUC‐MSCs/$2\times 10^{6}$ cells per kg/Intravenous	‐ The time to clinical improvement^b^ ‐ Improvement of: clinical symptoms of weakness and fatigue, shortness of breath, and low oxygen saturation‐ Creactive protein (CRP) and IL‐6^c^ ‐ The time for lymphocyte count returned to normal range was significant faster‐ Lung inflammation absorption was significantly shorter from CT imaging.	Shu et al. (2020)
COVID‐19 pneumonia/pilot clinical trial	ACE2‐ MSC/$1\times 10^{6}$ cells per kilogram of weight/intravenous	‐ Pulmonary function^e^ ‐ Peripheral lymphocytes^d^ (shift towards the regulatory phenotype for both CD4+ T cells and DCs)‐ Inflammatory cytokines^c^ ‐ IL‐10^d^ ‐ The CRP^b^	Leng et al. (2020)
ARDS treatment, a phase 1 clinical trial	BM‐MSC/$1\times 10^{6}$, $5\times 10^{6}$, $10\times 10^{6}$ cells per kg predicted bodyweight [PBW]/intravenous infusion/six months of follow‐up	‐ No prespecified infusion‐associated adverse events‐ Interleukin 6, 8^b^ ‐ ANGPT2 (angiopoietin‐2)^b^‐ AGER (receptor for advanced glycation endproducts)^b^	Wilson et al. (2015)
Treatment of ARDS	AS‐MSC/$1\times 10^{6}$ cells per kg of body weight/ntravenous infusion/28 days follow‐up	‐ No infusion toxicities or serious adverse events‐ Length of hospital stay, ventilator‐free days and ICU‐free days at Day 28 after treatment were similar. ‐ Serum SP‐D levels^c^ ‐ No significant changes in IL‐8 levels‐ The IL‐6 levels^b^ (but this trend was not statistically significant (*p* = .06)	Zheng, Huang et al. (2014)

*Note*: Clinical studies of this table include those that have published their outcomes.

Abbreviations: ALI, acute lung injury; BM‐MSC, bone marrow mesenchymal stem cell; COVID‐19, coronavirus disease 2019; CT, computed tomography; ICU, intensive care unit; IFN, interferon; IL, interleukin; KGF, keratinocyte growth factor; LPS, lipopolysaccharides; PDGF, platelet‐derived growth factor; SARS‐CoV‐2, severe acute respiratory syndrome coronavirus 2; TNF, tumor necrosis factor.

^a^ Prevention.

^b^ Decrease.

^c^ Significant decrease.

^d^ Enhancement or improvement.

^e^ Significant enhancement.

### 1.9 BM‐MSC

BM‐MSCs secrete various epithelial‐specific growth factors, particularly KGF (the fibroblast growth factor) that in pulmonary edema models it could decrease lung injury (Lee et al., 2011). IA endotoxin‐induced pulmonary edema can be returned to normal administering intrabronchial allogeneic BM‐MSC, in other words, the permeability of lung vascular and intravascular lung water ware affected by MSCs. It was shown that this is due to secretion of KGF. KGF influences the permeability of lung endothelial and epithelial to enhance the potential of alveolar epithelium to remove edema fluid of alveolar (Lee et al., 2009; Matthay et al., 2010). Trapped hBM‐MSCs after IV usage act as emboli in the mice lung and secreted TSG‐6 that is a powerful anti‐inflammatory factor TNF‐α‐induced protein 6. This finding proved the therapeutic role of hBM‐MSCs for related diseases (Lee et al., 2009). Anti‐inflammatory and reduced collagen deposition in mice challenged with BLM were reported in BM‐MSCs too (Ortiz et al., 2003). Mobilization of BM‐MSCs may be the general modulatory mechanism of acute inflammatory response. MSCs could moderate cytokine secretion of endotoxin injured lungs not only by humoral factor but also by operations that need direct contact of lung cells and stem cells (Xu et al., 2007). Xu et al. in 2008, showed the efficiency of MSC‐based Ang1 gene therapy acute lung injury treatment in mice (Xu et al., 2008).

Animal studies on pig and rodent using systemic BM‐MSC in influenza viruses (H5N1 and H7N9) could develop the dysregulated alveolar fluid clearance and protein permeability (Chan et al., 2016; Khoury et al., 2020). IV administration of murine BM‐MSCs in young immunocompetent mice model of avian influenza virus (H9N2)‐induced lung injury showed decreasing in lung edema, mortality and lung damage, enhancement in gas exchanging, as well as amount of anti‐inflammatory mediators but no reduction was seen in lung virus titration (Khoury et al., 2020; Li et al., 2016).

Investigating the effect of BM‐MSC on bronchopulmonary dysplasia (BPD) and emphysema of rat suggested that stem cell‐based treatments could suggest a new therapeutic approach for lung disorders (van Haaften et al., 2009). Another in vivo BPD study suggested that paracrine release of immune‐modulatory factors of BM‐MSCs could improve the vascular and parenchymal damage. They suggested that not only BM‐MSC but also its secretion factors could act as a new therapeutic strategies for lung disorders that right now there is no effectual treatments (Aslam et al., 2009).

Rao et al. in a clinical trial used allogeneic UC‐ or BM‐MSCs through IV route or intrapulmonary implantation to balance the neutralization of MSCs. They suggested cytokine cocktail derived from Th1 cells for MSC priming to overcome the hyperactive immune response as well as encourage tissue repair (Rao et al., 2020). Non‐immunosuppressed C57BL6 mice received BM‐MSC through the IT route to appraise the efficacy of MSC on lung injury. Higher Survival, lowered histologic lung injury, decreased pulmonary edema, pro‐inflammatory cytokines, and enhanced anti‐inflammatory cytokines were reported (Gupta et al., 2007; Matthay et al., 2010).

It was reported that mostly used MSC source for clinical trials for immune or inflammatory lung ailments is bone marrow (32.4%) (Yen et al., 2020). The potential of BM‐ and UC‐MSCs therapy in influenza virus‐induced lung injury in vitro and in vivo were gathered by Du et al. Results offered that MSCs are effective cell sources in treatment of influenza virus‐induced lung injury (Du et al., 2020).

### 1.10 UC‐MSC

Ease of isolation and culture, low immunogenicity, notable immune‐modulatory, and tissue repair activities, makes them an ideal choice for allogenic transfer therapy (Liang et al., 2020). After BM‐MSCs (32.4%), UC‐MSCs (29.4%) are the most common used source in clinical trials for immune or inflammatory lung diseases. In contrast, in clinical trials for COVID‐19, Umbilical cord is the most used source (32.3%) (Yen et al., 2020). Another recently published paper reported that 65% of MSCs used for COVID‐19 clinical trials were UC‐MSCs (Smith et al., 2020). This different can be related to the time of report and number of studies up to those days.

In A/H5N1‐associated acute lung damage, hUC‐MSCs showed a protective role. Whether the administration of hUC‐MSCs could lead to better clinical improvement or not was investigated, related results summarized in Table 1 (Shu et al., 2020).

A case report paper demonstrated using allogeneic hUC‐MSC for a critically affected COVID‐19 patient (3 days apart). Level of T cell become normal and the patient was not required a ventilator anymore and could walk and noticeable side effects were not seen (Liang et al., 2020; Metcalfe, 2020).

### 1.11 Menstrual‐blood‐derived MSCs (men‐MSCs)

The potential of source, painless noninvasive procedure, low immunogenicity, high proliferation capacity and free of ethical concerns make menstrual‐blood derived MSCs a suitable choice (Chen, Qu & Cheng, Chen, et al., 2019; Chen, Qu, & Xiang, 2019; Chen, Yu, et al., 2020; Khoury et al., 2014; Tang et al., 2020). Chen used Men‐MSCs for H7N9‐induced ARDS. Men‐MSCs transplantation notably enhances survival in preclinical and clinical investigations. In the five‐year follow‐up period, no adverse was observed. In their opinion, the pathological characteristics of SARS‐CoV‐2‐associated ARDS look similar to that of H7N9‐induced ARDS and MSC‐based treatment potentially could be an alternative for COVID‐19 treatment (Chen, Yu, et al., 2020).

Men‐MSCs could decrease inflammatory impact to defend cytokine storm in COVID‐19 patients. As an underlying mechanism, it was suggested that for the prevention of myofibroblasts activity, MSCs inhibited epithelia cell apoptosis and decrease inflammatory factor secretion. MSC therapy of COVID‐19 patients and especially those with ARDS or subsequent pulmonary fibrosis seems beneficial (Chen, Yu, et al., 2020).

### 1.12 Potential of MSC exosome and secretome in lung diseases and COVID‐19

As discussed previously, MSCs could secrete several paracrine factors that have ability to adjust endothelial and epithelial permeability, reduce inflammation, improve tissue repair, and restrain bacterial growth (Lee et al., 2011). These paracrine molecules and EVs play important role in the protective effect of stem cells. Protective effect of MSC‐EV in ARDS was indicated by different studies (Guglielmetti et al., 2020; Wu et al., 2018).

MSC‐EVs could be secrete from several sources such as adipose tissue, bone marrow, peripheral blood, amniotic fluid, umbilical cord, placenta, gingival tissues, and periodontal ligament (O'Driscoll, 2020). MSC‐EVs consist of various growth factors and cytokines, such as HGF, TGF‐β1, IL‐6, and IL‐10 (Burrello et al., 2016) and could transfer some molecules such as proteins, miRNA, and mRNA. Released exosomes from the endosomal compartment are known as being an integral element of the intercellular microenvironment. Limited antigenic components on the surface of MSC‐EVs make it a nonimmunogenic option (Biancone et al., 2012).

Taghavi et al. suggested MSCs‐derived exosomes as another option. They could utilize for the same immuno‐modulatory effect. The advantage is that there is no difficulty with cell maintenance and injection, ease access, phospholipid nature, and proper size. Their structure not only allows them to integrate the cell membrane but also preserves the contents of the exosomes from degradation (Taghavi‐Farahabadi et al., 2020). MSC‐EVs lead to comparable results and also more efficient than MSCs in bettering inflammation and damage in a range of in vivo lung damage models (Khoury et al., 2020). It is thought that EV therapy could be an alternative treatment to whole cell‐based therapy (Chrzanowski et al., 2020). In systemic administration, EVs may be lost or not reach the airways and lungs, so intranasal or inhalation delivery suggested as more attractive ways. Lack of self‐replicate in EVs, enhance their safety to avoid uncontrolled cell division (O'Driscoll, 2020). Generally, MSC‐EVs‐based therapies reported as efficient, quick, safe (Lanyu & Feilong, 2019), cost effective way with higher chance of long‐term storage, lower oncogenic and mutagenic risk, easier transportation, and better resistance to damage by microenvironment of adverse disease for treatment of lung injury and COVID‐19. Possibility of long‐term storage is a critical option for developing countries to use the therapy without needing sumptuous GMP manufacturing equipment (Askenase, 2020; Chrzanowski et al., 2020). Also, they could be more precisely standardized per dose and duration of biologic action, compared to the difficult variability of live MSC (Askenase, 2020). It was reported that not only MSC exosomes could be freeze‐dried (on‐site administration without refrigeration) but also could be freeze‐thawed (without toxic cryo‐preservatives) for months to years (Askenase, 2020; El Baradie et al., 2020).

**Figure 2:**
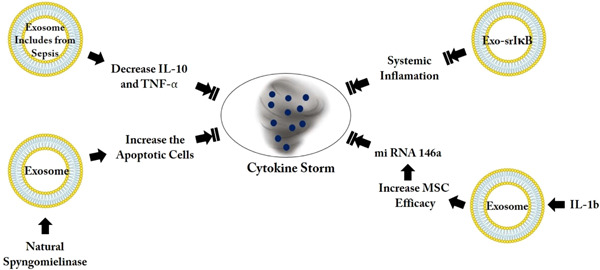
The main event of coronavirus disease 2019 (COVID‐19) infection and mesenchymal stromal cell (MSC)‐based treatment. In Step1, viruses are present in patient's lung and could infected others by dispersing of the virus particles (as an example by cough), then these particles enter into the body of healthy man (2), viruses bind to ACE2 receptor in cells of the respiratory system (3), symptoms could be different between patients from tolerable condition to intolerable states which the patient needs to be hospitalizing. These patients can make other people sick (4). MSC‐based therapies include MSCs, MSC‐EVs, and MSC‐secretome (5) that are from four main sources including, adipose, bone marrow, menstrual blood, and umbilical cord (6). (7) Results show the potential of these treatments in COVID‐19 recovery. (8) The recovered body could be infected and receive viruses again. EV, extracellular vesicle [Color figure can be viewed at wileyonlinelibrary.com]

It was approved that MSC‐ exosomes could facilitate oxygen exchange via enhancing of anti‐inflammatory mediators which could decrease lung injury intensity by enhancing alveolar epithelium permeability (Gupta et al., 2020; Worthington & Hagood, 2020). Inhalation administration of MSC‐exosomes could prevent exosome aggregation as reported in a pilot, clinical study (NCT04276987) (Chrzanowski et al., 2020). The possible effect of exosome on inhibition of the cytokine storm in the treatment of severe COVID‐19 is given in Figure 2. Reducing some cytokines (such as IL‐10 and TNF‐α) and enhancing the apoptosis of cells lead to reduction of systemic inflammation which all could cause by the exosomes. Also the exosomes could mediate miR‐146a regulation via MSC. Moreover purified srIkB‐loaded exosomes could reduce systemic inflammation and mortality of septic mouse models. All aspects of exosomes on treatment of COVID‐19 or its related symptoms is discuses on reference: (Kadriyan et al., 2020,). EVs interestingly not only can be useful for treatment but also could predict the severity of COVID‐19 (Fujita et al., 2020; Inal, 2020; Rosell et al., 2020).

Khatri et al. studied the effect of MSC‐EVs on lung epithelial cells. They found out MSC‐EVs not only transported the miRNAs, mRNAs and decrease the apoptosis but also inhibited the replication of influenza virus in cells. Furthermore they utilized a pig model infected by the influenza virus for in vivo study. Their outcomes confirmed that using MSC‐EVs result in reduction of virus replication, production of proinflammatory cytokines and finally lowered lung injury (Khatri et al., 2018; Taghavi‐Farahabadi et al., 2020). Also, Song et al. improved immuno‐modulatory effects of MSCs by pretreatment with IL‐1β. They suggested that this is due to the existence of exosomes that carry the miR‐146a to the cells (Song et al., 2017; Taghavi‐Farahabadi et al., 2020).

Papers indicated an inhibitory role in apoptosis of lung epithelial cells and replication of influenza virus (Khatri et al., 2018), attenuation of pulmonary inflammation, and increased airway hyperreactivity (BM) (Cruz et al., 2015). It was reported that exosomic vaccines including Spike proteins of SARS‐CoV cause highly neutralization of antibodies (Basiri et al., 2020; Kuate et al., 2007). This shows the potential of an exosome‐based vaccine for COVID‐19. Also as an adjunct or alternative, using ACE2+‐small MSC‐EVs considered as a inhibition therapy to reduce infection development (Inal, 2020). Urciuoli et al. suggested that as virally infected cells secured more EVs, inhibition of EV pathways seems to be an effective idea that needs to be investigated (Urciuoli & Peruzzi, 2020). There are nine clinical studies on clinicaltrial. gov till January 1st. Note that clinical trials of ARDS with MSC exosome have shown greater and more consistent benefit in part due to problems of the variability of viability and effects among the MSC when compared to MSC exosome, whereas the MSC exosome trials have been much more consistent (Askenase, 2020).

### 1.13 Exosomes derived from BM‐MSCs (BM‐MSC‐Exo)

Preclinical studies focusing on MSC‐EV efficiency on ALI and ARDS were gathered by Abraham et al. Results represent that till now, mostly investigated source of MSC‐EV was bone marrow. Using MSC‐EVs in clinic facing, some important challenges including variations in isolation, characterization, and purification of MSC‐EV that could cause highly heterogeneous EVs (Abraham & Krasnodembskaya, 2020).

In a mouse model of allergic airway inflammation that induced by the extract of Aspergillus Hyphal, systemic (via tail vein injection) usage of EVs and CM of BM‐MSCs (human and mouse) looks more beneficial and effective than their respective cells of source in decreasing the number of eosinophils and neutrophils. Additionally, EVs and CM of human MSC source seems more efficiency. Notwithstanding of difference between underlying inflammation models, anti‐inflammatory of MSC‐EVs is effective in more than one model of lung disease (Cruz et al., 2015).

BM‐MSC derived exosome therapy during hyperoxia indicated proangiogenic and anti‐inflammatory effects to preserve the lung from hyperoxia‐induced lung and BPD associated heart disease (Braun et al., 2018). Using hBM‐MSC‐EVs in a pulmonary fibrosis model that induced by bleomycin, showed that EVs could elevate anti‐inflammatory and immuno‐modulatory monocyte phenotype (Mansouri et al., 2019).

In a prospective cohort study, secreted exosomes (ExoFlo) of allogeneic BM‐MSCs were used through IV route for severe COVID‐19 treatment. Improvement of the clinical situation, neutrophil count, and oxygenation was observed after one treatment. Number of lymphopenia and lymphocytes increased and also reduced acute phase reactants were detected. The paper reported 83% survival rate. Results indicated recovery of 71% of patients, 13% of patients remained critically ill though stable. They reported safety, reconstitute immunity, restoring oxygenation capacity, downregulating of cytokine storm of this approach for severe COVID‐19 treatment (Sengupta et al., 2020).

### 1.14 AT‐MSCs derived exosomes (AT‐MSC‐Exo)

Various MiRs of fat tissue participated in adipokine secretion, adipogenesis regulation, intercellular communications, and inflammation. These present important insights in the role of AT‐MSC‐Exo in tissue and organs regeneration [18]. Gentile et al. suggested both autologous and allogeneic AT‐MSC, SVF, AT‐MSC Exosomal miRNA, and each type of MSCs as new substitute strategies for the COVID‐19 treatment (Gentile & Sterodimas, 2020).

### 1.15 UC‐MSCs derived EVs

Protective effects of intratracheally (IT) usage of hUC‐MSC versus hUC‐MSC‐EVs in lung injury of BPD rat model were studied by Porzionato et al. According to outcomes, both could decrease injuries induced by hypoxia but IT usage of MSC‐EVs seems efficacious in BPD treatment. As an example, MSC‐EVs could notably improve the total number of alveoli and a notable reduction in alveolar volume, whiles MSCs could significantly enhance the total number of alveoli only. Hyperoxia‐induced increasing of thickness in small pulmonary vessels could be significantly prevented by EVs only (Porzionato et al., 2019).

Although preliminary investigations showed the potential of MSC‐EV for COVID‐19 treatment, the scientific rationale for MSC‐EV administration and some issues needs to be well known, such as the source of MSC‐EVs, because EVs of a same tissue may demonstrate interindividual and probably clone‐specific functional diversities. Also, MSC‐EVs does not necessarily immune response suppressors but modulate it. To minimize the risk of side effects, the International Society for Cellular and Gene Therapies and the International Society for EVs recommend that the possible advantages and hazards of MSC‐EVs usage for COVID‐19 treatment should be weighed cautiously (Börger et al., 2020).

### 1.16 The secretome as “cell‐free” therapy

Different stresses such as acidosis, hypoxia, thermal stress, oxidative stress, and cytotoxic drugs could enhance vesicle liberation in cells. MSC secretome as paracrine factors of several bioactive molecules in therapeutic employment become interesting investigation (Khoury et al., 2014; Matthay et al., 2010). Using freeze‐drying technology to produce the powder of MSC secretome could be another treatment approach (Pourjabbar et al., 2020). It has previously proved that MSC secretome is a beneficial approach for pulmonary injuries of murine models. Deffune et al. suggested this approach for COVID‐19 patients in critical conditions (Deffune et al., 2020).

In BPD, MSC secretome similar to MSCs could modulate neonatal lung injury and also, maintain distal lung structure during period of lung development. Authors suggested that various issues need more investigation to find out more about CM that affect cells, or how exogenously applied MSCs act during hyperoxia (although endogenous MSC quantity in the blood and lung decrease due to hyperoxia, yet administration of a low amount of MSC could inhibit lung damage notwithstanding incessant exposure to hyperoxia) (Abman & Matthay, 2009).

The administration of secretome could have some issues, such as tumorigenicity, costs, immune incompatibility, and waiting for cell expansion which can be solved by using secretome. It has suggested that homeostasis, niche, and the physiological situation may affect the secretome signature (Khoury et al., 2014). In a perspective paper, MSC secretome offered as a new therapeutic strategy for COVID‐19 pneumonia. This is due to its extensive pharmacological impacts, such as immunomodulatory, proangiogenic, anti‐inflammatory, regenerative, and anti‐fibrotic properties (Bari et al., 2020).

### 1.17 Secretome of men‐MSCs

It was shown that Men‐MSCs could secrete angiogenic factors (ANG‐2, angiopoietin‐2), cytokine growth factors, such as PDGF‐BB, granulocyte‐macrophage colony‐stimulating factor, and matrix metalloproteinases (MMP3 and MMP10) much higher than UC‐MSCs (10–200,000 times). Nevertheless there was not difference in other angiogenic factors, such as EGF, HGF, and VEGF (Khoury et al., 2014).

### 1.18 LIFNano

In viral pneumonia, presence of leukemia inhibitory factor (LIF) to opposing the cytokine storm in the lung is necessary. MSC‐based LIF is not very cost‐effective, therefore, “LIFNano” as engineered stem cells showed 1000 times increase in potency. In vivo, results showed that it can be effective in the case of COVID‐19 pneumonia (Metcalfe, 2020).

#### 1.18.1 Bioactive lipids

It was shown that beneficial role of MSCS in COVID‐19 and other inflammatory diseases are related to their bioactive lipids (BALs) secretion, such as lipoxin A4 (LXA4), PGE2, etc (Das, 2020). Those MSCs with low capacity of BALs secretion are not able to perform their beneficial role well. Pretreatment of MSCs with BALs makes it possible to notable improves anti‐inflammatory effect of MSCs. It was suggested that BAL secretion (especially LXA4 and PGE2) by MSCs makes them efficient in COVID‐19 management. Due to the generation of proinflammatory TNF‐α and IL‐6 which could be inhibited by LXA4, PGE2, and their precursors (dihomo‐gamma‐linolenic acid, arachidonic acid, and gammalinolenic acid), they look useful for cytokine storm, immune checkpoint inhibitory therapy, ARDS, and sepsis (Das, 2020).

## 2 CONCLUSION

In the treatment of COVID‐19 patients, those approaches are a priority that could successful in related lung diseases such as ARDS, ALI, influenza and etc. In this article, cell‐based treatments and reports including various sources of MSC, MSC‐EVs, and other cellular products which were administrated for lung diseases especially coronavirus were reviewed. It is useful to gather repeated beneficial suggestions and experiments to get the ideal method and approach to improve symptoms and cure COVID‐19 patients.

Nearly all outcomes approved the beneficial effects of several sources of MSCs and their products. Although some products, such as secretome and EVs could be effective as well as MSCs. In fact, additional studies, such as cohort required to validate this therapeutic intervention further

## CONFLICT OF INTERESTS

The authors declare no competing interests.

## AUTHOR CONTRIBUTIONS

Conceptualization: Behnaz Banimohamad‐Shotorbani and Saeed Heidari keshel. Writing–Original Draft: Behnaz Banimohamad‐Shotorbani and Hekmat Farajpour. Writing–Review & Editing: Saeed Heidari keshel, Farshid Sefat, Shiva Ahdi Khosroshahi, and Hajar Shafaei. Supervision: Saeed Heidari keshel.
